# Supplementary material for: Evaluating the Use of Sacran, a Polysaccharide Isolated from Aphanothece sacrum, as a Possible Microbicide for Preventing HIV-1 Infection
Source: Viruses. 2024 Sep 23;16(9):1501. doi: 10.3390/v16091501 (PMC11437396; doi:10.3390/v16091501)
Supplement: Supplementary file 1 [file viruses-16-01501-s001.zip › viruses-3149599-supplementary.pdf]

Supplementary Figure S1

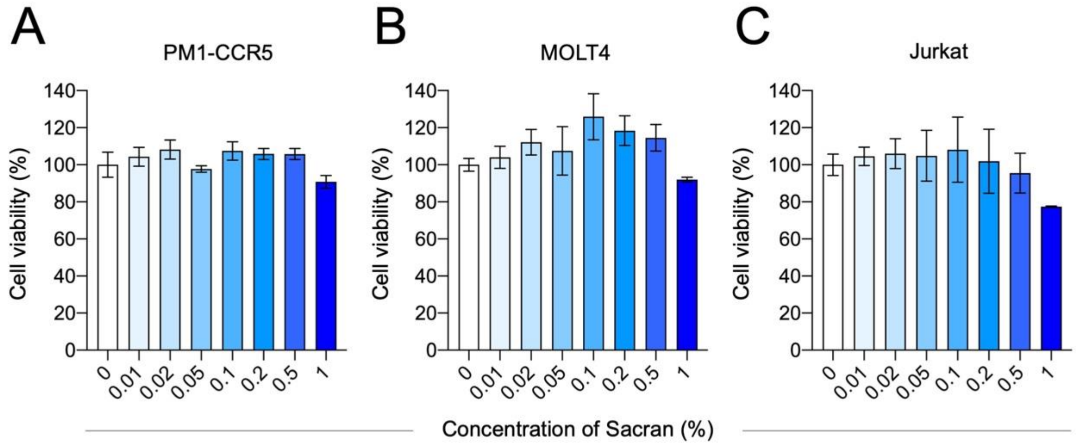

Cytotoxicity of Sacran gel

(**A**) PM1-CCR5 cells, (**B**) MOLT4 cells and (**C**) Jurkat cells were cultured in the presence or absence of sacran gel at concentration 0 to 1%. After 24 hours, cytotoxicity assay (WST-8 assay) was performed.
